# Thermal Analysis and Hybrid Compensation Design of a 10× Optical Zoom Periscope Lens for Smartphones

**DOI:** 10.3390/mi17010035

**Published:** 2025-12-28

**Authors:** Yi-Hong Liu, Chuen-Lin Tien, Yi-Lun Su, Wen-Shing Sun, Ying-Shun Hsu

**Affiliations:** 1 Department of Optics and Photonics, National Central University, Chungli 32001, Taiwan; yihongluis@gmail.com (Y.-H.L.); 112286002@cc.ncu.edu.tw (Y.-L.S.); wssun@dop.ncu.edu.tw (W.-S.S.); 108286005@cc.ncu.edu.tw (Y.-S.H.); 2 Department of Electrical Engineering, Feng Chia University, Taichung 40724, Taiwan

**Keywords:** periscope zoom lens, thermal analysis, hybrid thermal compensation, curved sensor

## Abstract

This study presents an optical and thermal design for a compact 10× periscope zoom lens suitable for smartphones, employing a hybrid thermal compensation scheme to ensure stable imaging performance over a wide range of temperatures. Our proposed zoom optics system integrates passive and active compensation mechanisms, further enhancing thermal stability through the use of a curved image sensor. Passive compensation is achieved through the selection of low-G optical materials and an optimized structural configuration. In contrast, active compensation dynamically adjusts the zoom group position in response to changes in ambient temperature. Optical simulations confirm that this 10× periscope zoom lens, composed of a prism, eight aspherical lenses, and two parallel plates, maintains diffraction-limited resolution and less than 2% distortion at all zoom positions (Zoom 1 to Zoom 6), achieving a total depth of 4.96 mm. Thermal analysis at temperatures ranging from −20 °C to 60 °C demonstrates that the optimized design, utilizing a curved sensor (Design type 3), achieves an average MTF of 0.58 and an average degradation rate of only 12.8%, exhibiting excellent non-thermal performance. These results highlight the effectiveness of the proposed novel hybrid thermal compensation strategy and surface sensor integration in realizing high-magnification, thermally stable periscope optics for next-generation smartphone imaging systems.

## 1. Introduction

With the increasing demand for ultra-thin form factors and high-resolution imaging in modern smartphones, conventional stacked-lens architectures have reached a practical limit in achieving high optical zoom. To overcome this constraint, periscope-type zoom lenses have been introduced, utilizing a 90° optical path deflection through a prism to allow the optical module to be arranged parallel to the smartphone plane. This configuration enables the integration of longer focal-length elements within compact device thickness, supporting high-magnification optical zoom without compromising slimness. Research by Park et al. [1,2,3] and Sun et al. [4,5] has shown the rapid evolution of periscope systems from conceptual prototypes to commercially viable designs. However, such high-precision, miniaturized optical assemblies are highly sensitive to temperature variations, as changes in refractive index and material expansion can cause focal shifts and aberration growth. These thermal drifts degrade imaging performance and necessitate precise thermal analysis and compensation during lens design.

Thermal effects in optical systems were first modeled by Grey [6], who analyzed temperature-dependent refractive index variation and its influence on focal plane stability. Since then, numerous athermalization techniques have been developed to mitigate temperature-induced aberrations. Passive approaches rely on material selection and structural balancing using elements with differing coefficients of thermal expansion (CTE) [7], while active systems employ temperature sensors and actuators to dynamically adjust lens positions [8]. Nasyrov [7] analyzed non-thermalization methods used in the optical systems of thermal imaging devices. Several passive non-thermalization methods were explored, involving the use of structural elements with different coefficients of linear thermal expansion at different temperatures. Park et al. [8] developed active athermalization systems for infrared lenses, where environmental temperature changes are detected by sensors. These changes are compensated for by adjusting the positions of the optical elements, representing an active form of thermal compensation. In contrast, passive thermal compensation typically takes two approaches: the first involves combining materials with different coefficients of thermal expansion (CTEs) or using specialized mechanical structures to minimize thermally induced positional shifts of optical components [9]. The second approach leverages the distinct thermo-optic properties of glasses to achieve material matching for athermal optical design [10,11]. Both methods aim to maintain image quality across wide thermal ranges, but achieving such stability in miniaturized zoom optics remains challenging.

In recent years, curved image sensors have emerged as a promising solution for both optical simplification and performance enhancement. Previous work by our team demonstrated that a curved image plane can significantly reduce field aberrations, improve edge illumination, and shorten lens length [12]. Furthermore, the inherent geometry of curved sensors naturally mitigates thermally induced focal shifts, offering new potential for passive thermal compensation [13]. In this study, we design a 10× periscope zoom lens for smartphones that combines passive and active thermal compensation mechanisms, while integrating a curved image sensor to further improve system stability. Optical simulations confirm that the proposed hybrid approach effectively maintains image quality across temperatures from −20 °C to 60 °C, meeting the stringent requirements of compact, high-performance smartphone camera modules.

## 2. Design Methods

Thermal compensation in optical systems refers to the techniques used to mitigate or eliminate the influence of temperature variations on optical performance through material selection, structural design, or active control. The goal is to maintain the stability of key optical parameters—such as focal length, aberrations, and image plane position—under fluctuating environmental temperatures. The underlying mechanisms primarily involve compensation of thermal expansion, refractive index variation, and optomechanical structural optimization. In general, passive athermalization is preferred for fixed-focus optical systems, while active compensation is more effective for zoom systems, where multiple movable groups allow dynamic adjustment. In this study, a hybrid approach is proposed: passive compensation forms the foundation of the design to address broad temperature variations, while active compensation provides fine-tuning for precision correction. Low-expansion optical materials with reduced thermo-optic constants (G-values) are used to minimize temperature sensitivity, and dynamic mechanical adjustments are applied to the zoom group to maintain optimal image quality.

### 2.1. Passive Compensation

The concept of passive thermal compensation lies in predefining low-expansion materials during the initial stage of optical system design, resulting in a relatively simple structural implementation. In a technical report by SCHOTT [14], the thermo-optical constant *G* (with units of K^−1^ or °C^−1^) is introduced to characterize the combined effect of thermal expansion (first term) and the thermo-optic effect (second term) on the refractive index, as expressed in Equation (1). Glasses with G-values approaching zero are referred to as athermal glasses, which are of critical importance for the design of high-precision optical systems, particularly under conditions where system performance must remain stable across varying temperatures.

(1) $$G=\alpha \cdot \left(n_{rel}\left(\lambda ,T\right)-1\right)+\frac{dn_{rel}(\lambda ,T)}{dT}$$

where α is the thermal expansion coefficient of the glass, describing the expansion or contraction of the material with temperature variation. $n_{rel}\left(\lambda ,T\right)$ represents the relative refractive index of the material at a specific wavelength, which is a function of both wavelength and temperature. $\frac{dn_{rel}(\lambda ,T)}{dT}$ denotes the derivative of the refractive index with respect to temperature, reflecting the thermo-optic coefficient of the material.

### 2.2. Active Compensation

Active thermal compensation introduces a closed-loop control mechanism to counteract temperature-induced optical deviations dynamically. This approach utilizes temperature sensors, actuators, and control algorithms to continuously adjust the positions of optical elements in response to environmental changes. Although it requires more complex hardware and software support, it provides greater precision and adaptability—especially for zoom lens systems where focal length and group spacing vary. In this design, the overall thermal behavior of the zoom system is first analyzed through optical simulation to determine the focal plane shift and MTF variation at different temperatures. Based on these results, fine positional adjustments are predefined for the zoom group as a function of temperature. During operation, the active compensation mechanism performs real-time corrections by slightly shifting the zoom group, thus maintaining athermalized performance over the full range from −20 °C to 60 °C. This hybrid thermal compensation strategy, which combines low-G-value glass selection with temperature-dependent mechanical actuation, forms the foundation for achieving high thermal stability in the proposed 10× periscope zoom lens.

## 3. Optical Design of a 10× Optical Zoom Periscope Lens

The proposed optical system is a compact 10× periscope zoom lens specifically designed for smartphone imaging modules. The design focuses on minimizing optical aberrations, maintaining image quality over the full zoom range, and ensuring thermal stability across varying environmental conditions. To achieve these objectives, the system integrates a right-angle prism for optical path folding, aspheric lens elements for aberration control, and a curved image sensor to further improve field curvature correction and reduce system length.

### 3.1. First-Order Design and Performance Requirements

For this study, we selected the OmniVision OV08X 9-megapixel CMOS (OmniVision Technologies, Santa Clara, CA, USA) image sensor due to its high resolution, pixel density, and availability in the market. This sensor has a 16:10 aspect ratio, and its key specifications are summarized in Table 1.

In this study, the semi-field angle is set at 22°. Using the relationship between image height, semi-field angle, and effective focal length (EFL), we calculated the EFL to be 3.96 mm. This paper discusses a 10× zoom lens system, dividing the EFL into six discrete zoom positions, labeled from Zoom 1 (wide-angle) to Zoom 6 (telephoto). Consequently, the EFL at the wide-angle end is 3.96 mm, while it reaches 39.60 mm at the telephoto end. The F-number (F/#) is determined by the EFL and the entrance pupil diameter (Den). For the wide-angle configuration, the F/# is set in the range of 3.2 to 11, with the entrance pupil diameter varying between 1.238 mm and 3.60 mm. The field of view is defined starting from a semi-field angle of 0° and an image height of 0 mm, referred to as Field 0, to a semi-field angle of 22.06° and an image height of 1.605 mm, designated as Field 11. To satisfy the design requirements, the image height is decomposed into X and Y directions. The first-order design parameters are summarized in Table 2.

This work establishes several key performance specifications for the designed optical system to ensure imaging quality meets application requirements. First, regarding the modulation transfer function (MTF), the system must achieve an MTF value greater than 0.5 at the design spatial frequency, corresponding to a diffraction-limited MTF of 0.75, across all zoom positions to ensure sufficient resolution performance. Second, lateral chromatic aberration (LCA) must be controlled to within one Airy disc diameter, defined as 2.44 × λ × F/#, to minimize color-induced degradation of image clarity. Third, optical distortion must be maintained below 2% across all zoom positions and field angles to ensure geometric accuracy is preserved. Fourth, relative illumination (RI) should exceed 70% at all zoom settings and field angles to avoid significant vignetting and loss of edge brightness. Finally, to accommodate the miniaturization requirements for integration into compact devices, such as smartphones, the total lens depth is constrained to be less than 5 mm. A summary of these design targets is provided in Table 3.

### 3.2. Result of Design Type 1 for a 10× Optical Zoom Periscope Lens

**Figure 1:**
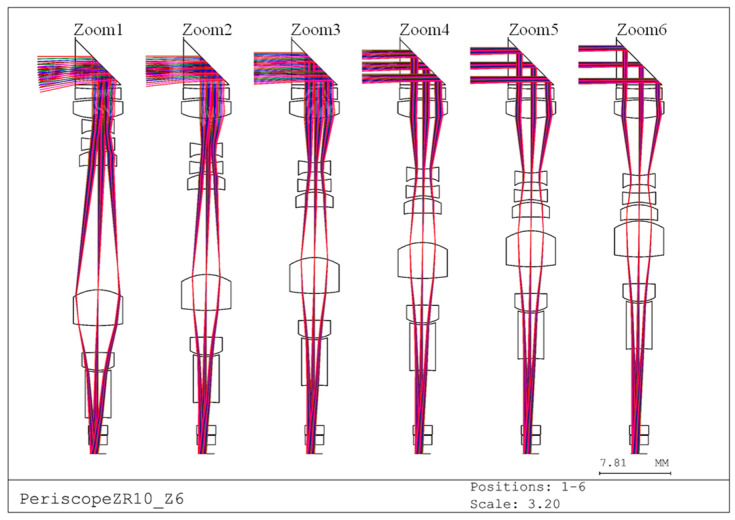
Plot of the lens configuration for a 10× periscope smartphone lens.

As shown in Figure 1, the first design is a 10× zoom, 9-megapixel periscope lens. From left to right, the configuration illustrates Zoom 1 (focal length: 3.96 mm) through Zoom 6 (focal length: 39.6 mm).

In this design, the lens depth is defined as the maximum aperture in the Y-direction from the first surface to the image plane of the periscope zoom lens (including the right-angle prism). To reduce the overall thickness of the lens module, the circular lenses in the original design were replaced with rectangular lenses, thereby decreasing the aperture size along the Y-direction. The viewing method was first employed to determine the effective aperture positions in the X- and Y-directions, followed by ray tracing to obtain numerical values at each position. These results were then used to specify the X- and Y-directional dimensions of each lens. The final lens depth was 4.96 mm. The zoom lens design includes lens parameters, zoom parameters, and aspheric coefficients as summarized in Table 4, Table 5, and Table 6, respectively. Notably, the air separations d7, d13, and d19 vary according to the zoom position. The configuration consists of one prism, three groups of eight aspheric lenses, and two plane-parallel plates (representing the IR-cut filter and protective glass), all of which are made of molded glass from SCHOTT [16]. Due to the 90° optical path deflection introduced by the prism, the sign of the curvature radii, lens thicknesses, air spaces, and higher-order aspheric coefficients beyond the third surface must be inverted.

### 3.3. Thermal Stability Analysis

In this study, two metrics are employed to evaluate the overall performance and thermal stability of the design: (1) analyzing the MTF values at every 10 °C increment within the −20 °C to 60 °C range, and calculating both the average MTF at each temperature and the overall average MTF to assess the optical performance; (2) evaluating the system’s thermal stability based on the variation trend of average MTF values under different temperature conditions. The MTF degradation ratio of the optical system is defined as:

(2) $$MTF\;degradation\;ratio=\frac{\left({MTF}_{best}-{MTF}_{T}\right)}{{MTF}_{best}}\times 100\%$$

where ${MTF}_{best}$ denotes the average MTF at the optimal temperature, and ${MTF}_{T}$ is the average MTF at another given temperature.

Table 7 presents the analysis of resolution performance (MTF values) of Design Type 1 over the temperature range from −20 °C to 60 °C. The design temperature is set at 22 °C, which is the default in the CODE V software (2023.03).

For Design Type 1, the best performance occurs within the room temperature range of 10 °C to 30 °C, specifically at 22 °C, where the average Modulation Transfer Function (MTF) across all zoom positions is 0.555. This value is defined as ${MTF}_{best}$, resulting in a degradation ratio of 0%. At low temperatures (−20 °C), the performance is poorest at Zoom 1, with an MTF value of 0.012. Additionally, the average MTF across all zoom positions drops to 0.346, leading to a degradation ratio of 37.7%. At high temperatures (ranging from 50 °C to 60 °C), the MTF gradually declines as well, reaching an average of 0.357 at 60 °C, which corresponds to a degradation ratio of 35.6%. Overall, when considering all environmental temperatures and zoom positions, Design Type 1 achieves an average MTF of 0.455, with a mean degradation ratio of 18%.

## 4. Athermalization Design of a 10× Optical Zoom Periscope Lens

### 4.1. Passive Thermal Compensation

#### 4.1.1. Design Type 2: Replacing Optical Glass with a Lower G-Value Material

To verify that optical glasses with lower thermo-optic constant (G) values reduce the thermal impact on the system, the initial step in the second design concept was to replace glasses with higher G-values with those with lower ones. Upon examination of the optical glasses used in Design Type 1, it was found that PSF68 exhibits a G-value as high as 32.669, while SF57 has a G-value of 19.597. Therefore, a replacement glass was sought with a similar refractive index and Abbe number, as well as being suitable for molding applications. Ultimately, PSF68 and SF57 were substituted with PSF67, which has a G-value of 10.311. The G-values of all optical materials in Design Type 2 are summarized in Table 8.

Design Type 2 continues to adopt the main framework of Design Type 1, with only four lenses (the 1st, 2nd, 4th, and 8th elements) replaced. After re-simulation and optimization, the system must also meet all optical quality requirements. Table 9 presents the analysis of resolution performance (MTF values) for Design Type 2 over the temperature range of −20 °C to 60 °C. The design temperature is set at 22 °C, which is the default in the CODE V software.

For Design Type 2, within the room-temperature range (10 °C to 30 °C), the best performance occurs at 22 °C, where the average MTF across all zoom positions is 0.576, defined as ${MTF}_{best}$, yielding a degradation ratio of 0. At low temperature (−20 °C), the poorest performance is observed at Zoom 1 with an MTF value of 0.009. At the same time, the average MTF across all zoom positions decreases to 0.36, corresponding to a degradation ratio of 37.4%. At high temperatures (50 °C to 60 °C), the MTF gradually declines, reaching an average value of 0.422 at 60 °C, with a degradation ratio of 26.6%. Overall, across all environmental temperatures and zoom positions, Design Type 2 achieves an average MTF of 0.485, with a mean degradation ratio of 15.8%.

#### 4.1.2. Design Type 3: Substitution with a Curved Image Sensor

Design Type 3 continues to utilize the lens materials from Design Type 2 and incorporates the first-order design from Design Type 1. The optimization process focuses on adjusting the curvature and thickness of the optical elements, the zoom parameters, and the curvature of the curved image sensor. After optimization, the radius of curvature (R) of the image surface is 16.374 mm, the diagonal length (D) of the sensor is 3.201 mm, and the ratio between them (D/R) is 0.195. This D/R ratio serves as a key parameter for evaluating the curvature of a curved image sensor, reflecting the influence of the sensor’s bending extent on the optical system design. According to literature and practical product data, the D/R ratio must be kept below 0.68; exceeding this value would significantly increase the manufacturing difficulty of the sensor and introduce unresolved challenges related to stress and material performance.

The results confirm that the implementation of active thermal compensation effectively enhances system performance. Table 10, Table 11, and Table 12, respectively, present the lens parameters, zoom parameters, and aspheric coefficients of Design Type 3 at 22 °C.

Table 13 presents the variations in resolving power (MTF values) for Design Type 3 across a temperature range of −20 °C to 60 °C, with evaluations taken at 10 °C intervals. The reference temperature of 22 °C is the default design temperature used in the CODE V software.

For Design Type 3, within the room-temperature range (10 °C to 30 °C), the best performance occurs at 22 °C, where the average MTF across all zoom positions is 0.665, defined as ${MTF}_{best}$, yielding a degradation ratio of 0. At high temperatures (50 °C to 60 °C), the poorest performance is observed at Zoom 1 with an MTF value of 0.125. Meanwhile, the average MTF across all zoom positions decreases to 0.471, corresponding to a degradation ratio of 29.3%. At low temperature (−20 °C), the average MTF value is 0.459, with a degradation ratio of 31%. Overall, across all environmental temperatures and zoom positions, Design Type 3 achieves an average MTF of 0.58, with a mean degradation ratio of 12.8%.

Based on the comparison between Design Type 1, Design Type 2, and Design Type 3, Design Type 3 has the highest average MTF value (0.580), followed by Design Type 2 (0.485), and the lowest is Design Type 1 (0.455), indicating that Design Type 1 has the best overall optical performance. In addition, Design Type 3 exhibits the smallest average MTF variation (12.8%), followed by Design Type 2 (15.8%), and the most significant variation is observed in Design Type 1 (18%). This indicates that Design Type 3 also provides the best temperature stability. The comparison of overall system performance among Design Types 1, 2, and 3 is summarized in Table 14.

**Figure 2:**
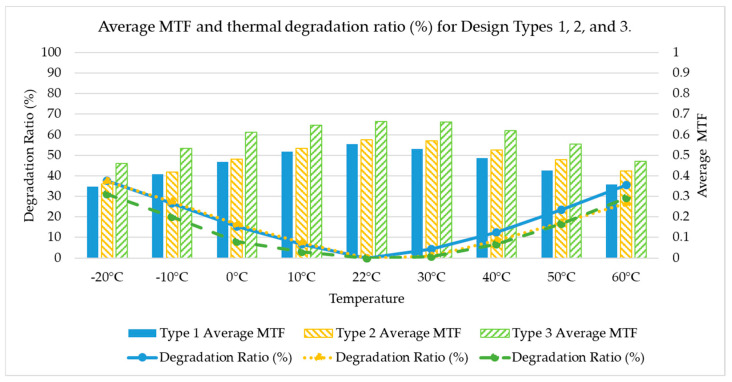
Average MTF and thermal degradation ratio (%) for Design Types 1, 2, and 3.

Figure 2 displays the average MTF and thermal degradation ratio of Design Types 1, 2, and 3 over a broad temperature range. In terms of MTF performance, Design Type 3 consistently exhibits the highest average MTF across all temperature points, clearly surpassing both Design Type 1 and Design Type 2. Regarding thermal degradation, Design Type 3 demonstrates the smallest reduction in MTF, while Design Type 1 suffers the most significant degradation. Furthermore, Design Type 3 maintains excellent optical consistency under both room-temperature and low-temperature conditions, and its MTF decay slope remains the flattest in the high-temperature region. These results collectively indicate that Design Type 3 delivers superior thermal stability and performance retention in wide-temperature applications.

### 4.2. Active Thermal Compensation

Based on the analysis of passive thermal compensation, Design Type 3 demonstrates superior optical performance and lower sensitivity to temperature variations. To further verify its robustness, active thermal compensation was applied to Design Type 3 under environmental conditions of −20 °C and 60 °C.

As shown in Table 15, when Design Type 3 is at −20 °C, the overall curvature and thickness of the lens system contract compared to those at 20 °C. Conversely, at 60 °C, the curvature and thickness expand relative to the values at 20 °C. This variation leads to defocus in the overall system. Here, d7, d13, and d19 are air separations that vary according to the zoom position, and they also exhibit thermal contraction or expansion at different temperatures. Only the conic constant remains unchanged.

The active thermal compensation mechanism is implemented through a pre-established lookup table (LUT). Its principle is based on predefining the positional adjustments of each lens group according to temperature variations. By means of the zoom mechanism, the system dynamically performs real-time correction to counteract aberrations induced by thermal effects. As a result, the positions of the lens groups vary not only with optical zoom but also undergo corresponding fine compensatory adjustments in response to different temperature conditions. Table 16 and Table 17 present the zoom parameters of Design Type 3 with active thermal compensation at −20 °C and 60 °C, respectively. After applying active thermal compensation, the minimum MTF value at −20 °C is 0.532, occurring at Zoom 6, Field 11. At 60 °C, the minimum MTF value after compensation is 0.579, occurring at Zoom 2, Field 11. A comparison of the MTF results is shown in Table 18.

## 5. Conclusions

This study presents a comprehensive optical and thermal design of a 10× periscope zoom lens optimized for smartphone imaging systems. The proposed architecture integrates a hybrid thermal compensation mechanism that combines passive and active correction, further enhanced by a curved image sensor to improve optical and thermal performance within an ultrathin form factor. Our design methodology is structured around an incremental progression through three configurations: a baseline (Design Type 1), a hybrid thermal compensation (Design Type 2), and a final design integrating a curved sensor (Design Type 3). This staged approach allows us to systematically deconvolve and evaluate the additive benefits of passive compensation, active compensation, and curved sensor technologies on the overall system performance.

In terms of thermal analysis and compensation, Design Type 3 had the highest average MTF of 0.58, followed by Design Type 2 (MTF = 0.485) and Design Type 1 (MTF = 0.455), indicating that Design Type 3 has the best overall optical performance. Furthermore, Design Type 3 exhibited the smallest mean degradation ratio of 12.8%, lower than Design Type 2 (15.8%) and Design Type 1 (18%), confirming that Design Type 3 also possesses excellent temperature stability. Simulation results show that the proposed periscope zoom lens design not only meets the requirements of a high zoom ratio and compact size but also achieves the expected performance in terms of image quality and thermal adaptability.

The adoption of a curved image sensor allows direct compensation for the inherent field curvature and substantially reduces optical distortion, thereby simplifying the lens assembly structure. Furthermore, this compensation lowers the sensitivity of residual aberrations to minute variations in inter-lens spacing. Consequently, the imaging performance of the system exhibits enhanced stability when temperature variations induce mechanical expansion or contraction. These results verify that the combination of low-G-value materials, active zoom-group correction, and curved sensor integration forms an effective pathway toward miniaturized, high-zoom, and thermally stable smartphone camera modules. Future work will focus on prototyping, tolerance analysis, and experimental validation of the proposed design to further assess manufacturability and integration feasibility in commercial mobile imaging systems.

## Figures and Tables

**Table 1:** Key specifications of the OmniVision OV08X CMOS image sensor [15].

Parameters	Specifications
Resolution	3840 × 2400 (9.2 Mega)
Aspect Ratio	16:10
Image Area	2.718 mm (X) × 1.707 mm (Y)
Pixel Size	0.7 μm × 0.7 μm
Diagonal	3.21 mm
Operating Temperature	−30 °C~+85 °C

**Table 2:** First-order design parameters for Design Type 1.

Zoom Position	Zoom Ratio	Focal Length (mm)	F/#	Semi-Field Angle (°)
Zoom 1	1×	3.96	3.2	22.06
Zoom 2	2×	11.88	6	7.69
Zoom 3	4×	19.8	7.5	4.63
Zoom 4	6×	27.72	9	3.31
Zoom 5	8×	35.64	10	2.58
Zoom 6	10×	39.6	11	2.32

**Table 3:** Lens design specifications.

Zoom Position	MTF	Lateral Color (Airy Disc)	Distortion	Relative Illumination
Zoom 1	105 lp/mm > 0.5	<4.583 μm	<2%	>70%
Zoom 2	56 lp/mm > 0.5	<8.594 μm	<2%	>70%
Zoom 3	45 lp/mm > 0.5	<10.742 μm	<2%	>70%
Zoom 4	37 lp/mm > 0.5	<12.891 μm	<2%	>70%
Zoom 5	34 lp/mm > 0.5	<14.323 μm	<2%	>70%
Zoom 6	30 lp/mm > 0.5	<15.755 μm	<2%	>70%

**Table 4:** Lens Parameters for Design Type 1 (Rectangular Lenses).

Surface No.	Surface Type	Radius (mm)	Thickness (mm)	Glass (SCHOTT)	Y Semi-Aperture	X Semi-Aperture
Object	Sphere	Infinity	Infinity			
1	Sphere	Infinity	2.500	PSF68	2.481	3.583
2	Sphere	Infinity	−2.500	PSF68	3.227	2.944
3	Sphere	Infinity	−0.457		2.280	2.776
4	Asphere	13.599	−1.000	PSF68	2.272	2.694
5	Asphere	−81.482	−0.200		2.297	2.659
6	Asphere	−9.882	−2.018	PLASF51	2.354	2.728
7	Asphere	10.190	d7		2.480	2.916
8	Asphere	3.624	−1.000	SF57	1.608	2.180
9	Asphere	−14.996	−0.928		1.342	1.727
10	Asphere	−30.428	−1.000	PLASF51	1.436	1.836
11	Asphere	−3.235	−0.302		1.661	2.136
12	Asphere	−3.823	−1.617	PSF67	1.853	2.339
13	Asphere	261.065	d13		1.746	2.154
STOP	Asphere	−4.810	−3.958	NFK51A	2.463	2.463
15	Asphere	14.166	−2.943		2.180	2.180
16	Asphere	−32.838	−1.952	NFK51A	1.605	1.737
17	Asphere	4.615	−0.200		1.431	1.619
18	Asphere	5.576	−5.000	PSF67	1.291	1.462
19	Asphere	−16.761	d19		1.005	1.297
20	Sphere	Infinity	−1.000	NBK7	0.961	1.306
21	Sphere	Infinity	−0.100		0.928	1.312
22	Sphere	Infinity	−1.000	NBK7	0.922	1.313
23	Sphere	Infinity	−1.000		0.889	1.327
Image	Sphere	Infinity	0.000		0.868	1.390

**Table 5:** Zoom Parameters for Design Type 1.

Parameters	Zoom 1	Zoom 2	Zoom 3	Zoom 4	Zoom 5	Zoom 6
EFL (mm)	−3.96	−7.92	−15.84	−23.76	−31.68	−39.60
F/#	3.2	6	7.5	9	10	11
d 7 (mm)	−0.351	−2.949	−4.908	−5.609	−6.037	−6.312
d13 (mm)	−13.69	−9.405	−5.584	−3.225	−1.512	−0.156
d19 (mm)	−0.901	−2.587	−4.449	−6.106	−7.392	−8.472

**Table 6:** Aspheric Coefficients for Design Type 1.

Surface	K	A	B	C	D	E
S_4_	−8.71 × 10^+1^	−2.67 × 10^−3^	4.32 × 10^−4^	−2.06 × 10^−5^	−1.43 × 10^−6^	1.45 × 10^−7^
S_5_	2.50 × 10^+1^	−6.17 × 10^−3^	7.99 × 10^−4^	−5.30 × 10^−5^	6.39 × 10^−7^	−1.00 × 10^−7^
S_6_	−1.56 × 10^+1^	1.44 × 10^−3^	−8.69 × 10^−5^	3.40 × 10^−5^	−1.99 × 10^−6^	−5.81 × 10^−8^
S_7_	7.08	2.27 × 10^−3^	−2.42 × 10^−4^	2.84 × 10^−5^	−1.50 × 10^−6^	3.10 × 10^−8^
S_8_	−1.18 × 10^+1^	−1.23 × 10^−2^	1.05 × 10^−3^	2.62 × 10^−5^	−1.11 × 10^−5^	4.83 × 10^−7^
S_9_	−7.42 × 10^+1^	−3.05 × 10^−2^	3.90 × 10^−3^	−1.50 × 10^−3^	1.25 × 10^−4^	−8.09 × 10^−6^
S_10_	−1.20 × 10^+1^	3.88 × 10^−2^	−8.57 × 10^−3^	4.51 × 10^−4^	1.66 × 10^−5^	−7.33 × 10^−16^
S_11_	−9.63	1.20 × 10^−2^	−1.99 × 10^−3^	3.31 × 10^−4^	−9.83 × 10^−6^	1.16 × 10^−12^
S_12_	−2.07	3.56 × 10^−4^	−3.72 × 10^−4^	5.66 × 10^−6^	6.41 × 10^−7^	8.78 × 10^−10^
S_13_	−9.91 × 10^−1^	−4.23 × 10^−3^	−5.47 × 10^−4^	−2.69 × 10^−5^	2.42 × 10^−6^	−9.54 × 10^−12^
S_14_	−1.10	−5.28 × 10^−4^	−4.97 × 10^−5^	1.25 × 10^−5^	−1.55 × 10^−6^	8.27 × 10^−8^
S_15_	−9.90 × 10^+1^	2.76 × 10^−3^	−8.99 × 10^−4^	1.95 × 10^−4^	−2.34 × 10^−5^	1.20 × 10^−6^
S_16_	−9.90 × 10^+1^	−3.03 × 10^−3^	1.15 × 10^−3^	−3.01 × 10^−5^	3.79 × 10^−5^	1.05 × 10^−6^
S_17_	1.64	8.06 × 10^−3^	−5.54 × 10^−4^	2.07 × 10^−4^	−4.66 × 10^−5^	1.39 × 10^−6^
S_18_	4.13	8.23 × 10^−3^	−1.45 × 10^−3^	1.55 × 10^−4^	−6.22 × 10^−5^	2.14 × 10^−15^
S_19_	1.85 × 10^+1^	2.52 × 10^−3^	−8.92 × 10^−4^	2.00 × 10^−4^	−3.56 × 10^−5^	−3.65 × 10^−17^

**Table 7:** Temperature Analysis for Design Type 1.

Zoom Position	−20 °C	−10 °C	0 °C	10 °C	22 °C	30 °C	40 °C	50 °C	60 °C
Zoom 1	0.012	0.062	0.173	0.320	0.508	0.406	0.267	0.134	0.048
Zoom 2	0.474	0.520	0.558	0.564	0.561	0.551	0.531	0.486	0.426
Zoom 3	0.450	0.521	0.562	0.559	0.539	0.516	0.474	0.424	0.368
Zoom 4	0.408	0.478	0.539	0.585	0.590	0.579	0.55	0.505	0.426
Zoom 5	0.334	0.404	0.469	0.526	0.561	0.569	0.559	0.529	0.474
Zoom 6	0.396	0.460	0.514	0.550	0.568	0.564	0.530	0.472	0.400
Average	0.346	0.408	0.469	0.517	0.555	0.531	0.485	0.425	0.357
Degradation Ratio (%)	37.7	26.5	15.4	6.7	0.0	4.3	12.5	23.4	35.6

**Table 8:** Thermo-Optic Constants (Low G-value) of Optical Materials in Design Type 2.

Surface No.	Glass (SCHOTT)	n_e_	V_e_	Relative Temperature Coefficients of Refractive Index [+20/+40 deg. °C], e-Line [10^−6^ K]	Alpha −30/70 [10^−6^ K]	G-Value [10^−6^ K]
1, 2, 4	PSF68	2.016	20.82	24.1	8.43	32.67
6, 10	PLASF51	1.815	40.68	8.7	6.01	13.60
8	SF57	1.855	23.64	12.5	8.3	19.60
12, 18	PSF67	1.917	21.23	4.6	6.23	10.31
14, 16	NFK51A	1.488	84.07	−4.6	12.74	1.62
20, 22	NBK7	1.519	63.96	3	7.1	6.68

**Table 9:** Temperature Analysis for Design Type 2.

Zoom Position	−20 °C	−10 °C	0 °C	10 °C	22 °C	30 °C	40 °C	50 °C	60 °C
Zoom 1	0.009	0.064	0.189	0.338	0.504	0.453	0.297	0.147	0.057
Zoom 2	0.268	0.348	0.431	0.489	0.523	0.539	0.534	0.511	0.480
Zoom 3	0.487	0.553	0.603	0.603	0.587	0.565	0.528	0.480	0.417
Zoom 4	0.467	0.526	0.578	0.620	0.652	0.668	0.612	0.570	0.515
Zoom 5	0.442	0.491	0.536	0.573	0.604	0.613	0.612	0.592	0.541
Zoom 6	0.488	0.522	0.549	0.569	0.584	0.583	0.575	0.559	0.524
Average	0.360	0.417	0.481	0.532	0.576	0.570	0.526	0.477	0.422
Degradation Ratio (%)	37.4	27.5	16.4	7.6	0.0	1.0	8.6	17.2	26.6

**Table 10:** Lens Parameters for Design Type 3 (Rectangular Lenses).

Surface No.	Surface Type	Radius (mm)	Thickness (mm)	Glass (SCHOTT)	Y Semi-Aperture	X Semi-Aperture
Object	Sphere	Infinity	Infinity			
1	Sphere	Infinity	2.500	PSF67	2.500	3.614
2	Sphere	Infinity	−2.500	PSF67	3.249	2.941
3	Sphere	Infinity	−0.494		2.295	2.763
4	Asphere	32.742	−1.000	PSF67	2.287	2.677
5	Asphere	−14.573	−0.227		2.344	2.746
6	Asphere	−10.544	−1.813	PLASF51	2.391	2.801
7	Asphere	8.965	d7		2.500	2.957
8	Asphere	3.717	−1.000	PSF67	1.611	2.188
9	Asphere	−20.170	−0.939		1.306	1.682
10	Asphere	−59.178	−1.139	PLASF51	1.410	1.798
11	Asphere	−2.899	−0.301		1.675	2.154
12	Asphere	−3.375	−1.666	PSF67	1.895	2.387
13	Asphere	217.316	d13		1.766	2.180
STOP	Asphere	−4.541	−3.444	NFK51A	2.500	2.377
15	Asphere	14.542	−2.908		2.204	2.216
16	Asphere	33.883	−1.392	NFK51A	1.598	1.711
17	Asphere	4.018	−0.210		1.540	1.704
18	Asphere	6.597	−5.000	PSF67	1.335	1.480
19	Asphere	−11.182	d19		1.013	1.279
20	Sphere	Infinity	−1.000	NBK7	0.958	1.298
21	Sphere	Infinity	−0.100		0.927	1.309
22	Sphere	Infinity	−1.000	NBK7	0.923	1.311
23	Sphere	Infinity	−1.000		0.892	1.321
Image	Sphere	−16.347	0.000		0.871	1.381

**Table 11:** Zoom Parameters for Design Type 3.

Parameters	Zoom 1	Zoom 2	Zoom 3	Zoom 4	Zoom 5	Zoom 6
EFL (mm)	−3.96	−7.92	−15.84	−23.76	−31.68	−39.60
F/#	3.20	6.00	7.50	9.00	10.00	11.00
d7 (mm)	−0.33	−2.93	−4.93	−5.63	−6.06	−6.35
d13 (mm)	−13.81	−9.54	−5.73	−3.35	−1.61	−0.20
d19 (mm)	−1.22	−2.90	−4.70	−6.38	−7.69	−8.81

**Table 12:** Aspheric Coefficients for Design Type 3.

Surface	K	A	B	C	D	E
S_4_	9.89 × 10^1^	−1.57 × 10^−3^	3.57 × 10^−4^	−3.01 × 10^−6^	−3.76 × 10^−6^	2.24 × 10^−7^
S_5_	−9.90 × 10^1^	−4.70 × 10^−3^	8.27 × 10^−4^	−5.46 × 10^−5^	1.67 × 10^−6^	−1.69 × 10^−7^
S_6_	−1.21 × 10^1^	2.08 × 10^−3^	−2.53 × 10^−4^	4.72 × 10^−5^	−5.60 × 10^−7^	−1.91 × 10^−7^
S_7_	5.28	1.50 × 10^−3^	−2.33 × 10^−4^	2.26 × 10^−5^	−3.99 × 10^−7^	−3.01 × 10^−8^
S_8_	−1.47 × 10^1^	−1.71 × 10^−2^	1.75 × 10^−3^	−1.86 × 10^−5^	−9.69 × 10^−6^	3.26 × 10^−7^
S_9_	9.90 × 10^1^	−3.54 × 10^−2^	3.82 × 10^−3^	−1.97 × 10^−3^	2.61 × 10^−4^	−8.09 × 10^−6^
S_10_	−1.16 × 10^1^	4.00 × 10^−2^	−1.18 × 10^−2^	1.27 × 10^−3^	−4.51 × 10^−5^	−1.99 × 10^−13^
S_11_	−8.37	1.13 × 10^−2^	−1.53 × 10^−3^	2.77 × 10^−4^	−8.48 × 10^−6^	8.46 × 10^−9^
S_12_	−2.72	−6.73 × 10^−4^	−3.88 × 10^−4^	−8.39 × 10^−6^	2.76 × 10^−6^	−1.30 × 10^−8^
S_13_	−9.67 × 10^1^	−5.48 × 10^−3^	−5.26 × 10^−4^	−6.72 × 10^−5^	8.67 × 10^−6^	2.95 × 10^−8^
S_14_	−1.19	−4.22 × 10^−4^	−3.67 × 10^−5^	−3.67 × 10^−5^	−1.13 × 10^−6^	7.79 × 10^−8^
S_15_	−9.90 × 10^1^	3.56 × 10^−3^	−8.80 × 10^−4^	1.86 × 10^−4^	−2.20 × 10^−5^	1.20 × 10^−6^
S_16_	−9.90 × 10^1^	5.41 × 10^−4^	1.01 × 10^−3^	6.12 × 10^−5^	1.04 × 10^−4^	1.05 × 10^−6^
S_17_	6.95 × 10^−1^	8.30 × 10^−3^	1.51 × 10^−3^	−4.42 × 10^−4^	4.16 × 10^−5^	1.39 × 10^−6^
S_18_	1.58	1.06 × 10^−2^	9.12 × 10^−4^	−1.25 × 10^−4^	−1.33 × 10^−4^	1.56 × 10^−15^
S_19_	4.09 × 10^1^	5.89 × 10^−3^	9.81 × 10^−4^	−3.64 × 10^−5^	6.26 × 10^−5^	−2.56 × 10^−15^

**Table 13:** Temperature Analysis for Design Type 3.

Zoom Position	−20 °C	−10 °C	0 °C	10 °C	22 °C	30 °C	40 °C	50 °C	60 °C
Zoom 1	0.181	0.335	0.501	0.634	0.673	0.657	0.525	0.323	0.125
Zoom 2	0.515	0.560	0.586	0.601	0.603	0.599	0.581	0.552	0.510
Zoom 3	0.526	0.570	0.697	0.634	0.645	0.643	0.621	0.581	0.523
Zoom 4	0.515	0.584	0.643	0.680	0.697	0.698	0.676	0.633	0.565
Zoom 5	0.487	0.557	0.616	0.662	0.693	0.695	0.671	0.624	0.553
Zoom 6	0.528	0.585	0.631	0.664	0.680	0.676	0.653	0.613	0.547
Average	0.459	0.532	0.612	0.646	0.665	0.661	0.621	0.554	0.471
Degradation Ratio (%)	31.0	20.0	7.9	2.9	0.0	0.6	6.6	16.7	29.3

**Table 14:** Comparison of Lens Performance between Design Types 1, 2, and 3.

Parameters	Design Type 1	Design Type 2	Design Type 3
Lateral color	Zoom1: 1.82 μm	Zoom1: 1.1 μm	Zoom1: 0.61 μm
	Zoom2: 3.04 μm	Zoom2: 2.98 μm	Zoom2: 2.94 μm
	Zoom3: 5.42 μm	Zoom3: 3.25 μm	Zoom3: 3.17 μm
	Zoom4: 5.93 μm	Zoom4: 2.8 μm	Zoom4: 2.74 μm
	Zoom5: 6.02 μm	Zoom5: 2.77 μm	Zoom5: 2.31 μm
	Zoom6: 6.1 μm	Zoom6: 2.57 μm	Zoom6: 2.15 μm
Relative illuminance	85.26%	86.35%	82.13%
Lens depth	4.96 mm	5 mm	5 mm
Average MTF Values of Each Zoom Position at 22 °C	0.555	0.576	0.665
Average MTF Values of Each Zoom Position from −20 °C to 60 °C	0.455	0.485	0.58
MTF Degradation Ratio of Each Zoom Position from −20 °C to 60 °C	18%	15.8%	12.8%

**Table 15:** Lens Parameters for Design Type 3 at −20 and 60 °C.

Surface No.	Surface Type	Radius (mm)		Thickness (mm)		Glass (SCHOTT)
		−20 °C	60 °C	−20 °C	60 °C	
Object	Sphere	Infinity	Infinity	Infinity	Infinity	
1	Sphere	Infinity	Infinity	2.499	2.500	PSF67
2	Sphere	Infinity	Infinity	−2.499	−2.500	PSF67
3	Sphere	Infinity	Infinity	−0.494	−0.494	
4	Asphere	32.734	32.750	−1.000	−1.000	PSF67
5	Asphere	−14.569	−14.576	−0.228	−0.227	
6	Asphere	−10.541	−10.546	−1.812	−1.813	PLASF51
7	Asphere	8.963	8.967	d7		
8	Asphere	3.716	3.718	−1.000	−1.000	PSF67
9	Asphere	−20.165	−20.175	−0.938	−0.939	
10	Asphere	−59.163	−59.192	−1.139	−1.139	PLASF51
11	Asphere	−2.898	−2.899	−0.303	−0.300	
12	Asphere	−3.374	−3.376	−1.666	−1.666	PSF67
13	Asphere	217.261	217.369	d13		
STOP	Asphere	−4.539	−4.543	−3.442	−3.446	NFK51A
15	Asphere	14.534	14.549	−2.905	−2.910	
16	Asphere	33.865	33.900	−1.391	−1.392	NFK51A
17	Asphere	4.016	4.020	−0.210	−0.210	
18	Asphere	6.595	6.598	−4.999	−5.001	PSF67
19	Asphere	−11.179	−11.184	d19		
20	Sphere	Infinity	Infinity	−1.000	−1.000	NBK7
21	Sphere	Infinity	Infinity	−0.100	−0.100	
22	Sphere	Infinity	Infinity	−1.000	−1.000	NBK7
23	Sphere	Infinity	Infinity	−0.999	−1.001	
Image	Sphere	−16.347	−16.347	0.000	0.000	

**Table 16:** Zoom Parameters for Design Type 3 at −20 °C.

Parameters	Zoom 1	Zoom 2	Zoom 3	Zoom 4	Zoom 5	Zoom 6
**EFL (mm)**	−3.96	−7.92	−15.84	−23.76	−31.68	**−39.60**
**F/#**	3.20	6.00	7.50	9.00	10.00	**11.00**
**d7 (mm)**	−0.332	−2.937	−4.900	−5.620	−6.056	**−6.311**
**d13 (mm)**	−13.755	−9.485	−5.658	−3.286	−1.544	**−0.200**
**d19 (mm)**	**−1.208**	**−2.873**	**−4.737**	**−6.389**	**−7.695**	**−8.784**

**Table 17:** Zoom Parameters for Design Type 3 at 60 °C.

Parameters	Zoom 1	Zoom 2	Zoom 3	Zoom 4	Zoom 5	Zoom 6
**EFL (mm)**	−3.96	−7.92	−15.84	−23.76	−31.68	**−39.60**
**F/#**	3.20	6.00	7.50	9.00	10.00	**11.00**
**d7 (mm)**	−0.363	−2.947	−4.905	−5.624	−6.059	**−6.351**
**d13 (mm)**	−13.896	−9.608	−5.785	−3.417	−1.680	**−0.272**
**d19 (mm)**	**−1.223**	**−2.925**	**−4.791**	**−6.440**	**−7.743**	**−8.858**

**Table 18:** MTF of Design Type 3 before and after active thermal compensation.

	−20 °C		60 °C	
	Before	After	Before	After
Zoom 1	0.181	0.627	0.125	0.641
Zoom 2	0.515	0.555	0.51	0.579
Zoom 3	0.526	0.589	0.523	0.594
Zoom 4	0.515	0.665	0.565	0.659
Zoom 5	0.487	0.669	0.553	0.648
Zoom 6	0.528	0.532	0.547	0.626

## Data Availability

The original contributions presented in this study are included in the article. Further inquiries can be directed to the corresponding author.
